# Mouse models for preeclampsia: disruption of redox-regulated signaling

**DOI:** 10.1186/1477-7827-7-4

**Published:** 2009-01-15

**Authors:** Subhasis Banerjee, Harpal Randeva, Anne E Chambers

**Affiliations:** 1 Clinical Sciences Research Institute, Medical School Building, Gibbet Hill Campus, University of Warwick, Coventry CV4 7AL, UK

## Abstract

The concept that oxidative stress contributes to the development of human preeclampsia has never been tested in genetically-defined animal models. Homozygous deletion of catechol-O-methyl transferase (Comt-/-) in pregnant mice leads to human preeclampsia-like symptoms (high blood pressure, albuminurea and preterm birth) resulting from extensive vasculo-endothelial pathology, primarily at the utero-fetal interface where maternal cardiac output is dramatically increased during pregnancy. Comt converts estradiol to 2-methoxyestradiol 2 (2ME2) which counters angiogenesis by depleting hypoxia inducible factor-1 alpha (HIF-1 alpha) at late pregnancy. We propose that in wild type (Comt++) pregnant mice, 2ME2 destabilizes HIF-1 alpha by inhibiting mitochondrial superoxide dismutase (MnSOD). Thus, 2ME2 acts as a pro-oxidant, disrupting redox-regulated signaling which blocks angiogenesis in wild type (WT) animals in physiological pregnancy. Further, we suggest that a lack of this inhibition under normoxic conditions in mutant animals (Comt-/-) stabilises HIF-1 alpha by inactivating prolyl hydroxlases (PHD). We predict that a lack of inhibition of MnSOD, leading to persistent accumulation of HIF-1 alpha, would trigger inflammatory infiltration and endothelial damage in mutant animals. Critical tests of this hypothesis would be to recreate preeclampsia symptoms by inducing oxidative stress in WT animals or to ameliorate by treating mutant mice with Mn-SOD-catalase mimetics or activators of PHD.

## Background

Approximately 5–7% of pregnant women worldwide suffer from common hypertensive pregnancy disorders culminating in preeclampsia (PE), intrauterine growth restriction and premature child birth. PE is the major cause of maternal mortality (80%) in developing nations and in recent years, the perinatal mortality and morbidity in developed countries have increased by five-fold [1,2]. Moreover, the incidence of PE has increased by 40% in the last 15 years [3]. The most widely accepted cause of pre-eclampsia is reduced utero-placental circulation (superficial implantation of the fetus) due to sub-optimal vascular remodeling of the decidual and the uterine arterioles, secondary to inadequate trophoblast invasion [4]. Increased oxidative stress and an altered immune response [5] at the fetal-maternal interface (Th1 bias) are likely effectors contributing to the development of systemic endothelial and renal dysfunctions in the later phase of the disease.

**Figure 1:**
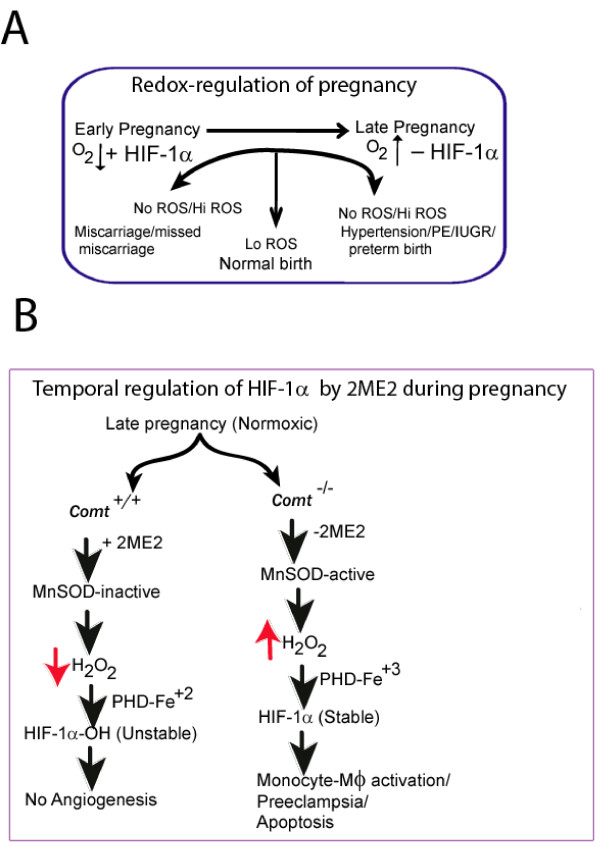
**Redox-regulated signaling in normal pregnancies and in Comt-deficient mutant mice**. A, Shows that ROS-induced signaling (Lo ROS) is essential for implantation, establishment and maintenance of pregnancy. The absence of ROS (No ROS) or its excessive accumulation (Hi ROS) are detrimental to pregnancy; B, in *Comt*^+/+ ^mice (WT), 2ME2 production is highest at late pregnancy and blocks vascular growth by destabilizing HIF-1α in physiological pregnancies. The absence of 2ME2 in *Comt*^-/- ^animals (mutant) would increase oxidative stress and stabilize HIF-1α. The stability of HIF-1α is dependent upon critical concentration of H_2_O_2 _which determines the functional state of prolyl hydroxylases (PHD).

A series of recent discoveries, specifically the isolation and functional characterization of non-phagocytic NADPH oxidase-homologues in epithelial, endothelial, fibroblast and muscle cells, argue that reactive oxygen species (ROS) are indispensible to both physiological and patho-physiological conditions such as growth, differentiation, apoptosis and senescence [6-8]. The transition from growth to degeneration is finely tuned by the relative concentration of oxidants. For example, in ambient conditions, low levels of H_2_O_2 _(nano-micromolar) are necessary for angiogenesis [9], while agonist-induced activation resulting in its excessive accumulation (> 150–200 μM) prompts endothelial damage [10]. The scheme shown in Fig. 1a depicts the essential role played by ROS in both early and late pregnancy together with the detrimental effect when ROS are produced in excess (oxidative stress).

During pregnancy, the maternal energy demand increases significantly to sustain the growing fetus. This demand is met via a substantial increase (30–50% compared to the non-gravid state) in the uterine blood flow in the pregnant women [11]. Consequently the 'shear stress' (dragging frictional force generated by the blood flow), is negotiated by endothelial cell (EC)-derived vasodilatory agonists, nitric oxide (NO^.^) and prostaglandin I_2_. The release of vasodilators from EC is controlled by mitochondrial Ca^+2^-influx (ATP-GPCR and ISP3-ER pathways) as well as NADPH oxidase-dependent mitochondrial O_2_^.- ^production. The pregnancy-induced adaptation of ROS-regulated Ca^+2 ^signaling in the mitochondria of EC is essential for re-establishing physiological laminar flow in uterine vessels [12]. In extreme circumstances, dysregulation of mitochondrial Ca^+2 ^homeostasis due to high ROS, could lead to Ca^+2 ^overload of the matrix, triggering apoptosis. The first indication of a mitochondrial involvement in preeclampsia/eclampsia came from a case report where the frequency of the disease was high in a family with inborn mitochondrial defects [13]. A number of *in vitro *studies on a hypoxia-reoxygenation model of placental culture and plasma oxidant/antioxidant analyses further pointed to mitochondrial involvement by suggesting that ROS could influence trophoblast fusion, migration and apoptosis relevant to preeclampsia [14,15]. In support of this, it is pertinent that homozygous deletion of *SOD-2 *(MnSOD) has the most severe effect on embryonic development in mouse pregnancy compared to that of *SOD-1 *and *SOD-3 *knockouts [16].

Reactive oxygen species (ROS) are key mediators of growth factor-dependent redox-regulated signaling in angiogenesis. While > 90% of O_2 _is reduced in mitochondria, NADPH oxidases (Nox2, gp91phox) are the major source of ROS in endothelial cells [17,18]. The O_2_^.- ^generated at the outer surface of the plasma membrane is internalized through ion channels, a process which increases the intracellular Ca^+2 ^release, activating mitochondrial O_2_^.- ^production. Thus, NADPH oxidases and mitochondria together perpetuate a cascade of O_2_^.- ^production in vascular endothelial cells [19,20].

Hypoxia inducible factor -1α(HIF-1α) is a dynamic partner of the heterodimeric transcription factor HIF-1 which is essential for angiogenesis [21]. Irrespective of the oxygen tension (hypoxic or normoxic), the stability of HIF-1α is determined by it ligation to von Hippel-Lindau tumor suppressor protein, VHL [22]. Once bound to VHL, HIF-1α undergoes ubiquitination prior to proteosomal degradation. A prerequisite for VHL binding is site-specific hydroxylation of HIF-1α by prolyl hydroxylases (PHD). The transfer of these hydroxyl moieties to HIF-1α by PHD requires O_2 _and 2-oxogluterate as co-substrates, together with reduced iron (Fe^+2^) and ascorbate as cofactors [23,24]. Therefore, prolyl hydroxylases act as negative regulators of HIF-1α since active PHD-Fe^+2 ^promote HIF-1α binding to VHL and subsequent degradation. It is apparent that the enzymatic activity of PHD could be modulated by the relative concentration of co-substrates as well as cofactors. Indeed, the stability of HIF-1α in angiotensin II-treated vascular smooth muscle cells under normoxic conditions is due to H_2_O_2_-mediated reduction in cellular ascorbate concentration and increased Fe^+3 ^[25]. It is noteworthy that commonly encountered ROS such as O_2_^.- ^and OH^. ^anions are short-lived and, owing to their limited diffusion capacity, fail to cross the plasma membrane except through ion channels. Therefore, the majority of cellular ROS-effects are mediated via relatively stable H_2_O_2_. This is consistent with genetic, molecular and pharmacological experiments [26-29] suggesting that H_2_O_2 _plays a central role in stabilizing HIF-1α by converting PHD-Fe^+2 ^to PHD-Fe^+3^.

Clinical research on preeclampsia (PE) is mostly restricted to observations, *in vitro *correlative studies on placenta and patient serum samples originating from mid-to-late gestation when the disease is fully manifested, long after it initiates at early pregnancy. These problems have underlined the necessity for animal models of PE where the progression of pathogenesis can be traced through longitudinal studies from early pregnancy. Three recently developed mouse models for PE [30-32] exhibit hallmarks of the human disease. In one of these models, the critical symptoms of PE (hypertension, proteinurea due to glomerulosclerosis and fetal resorption) were ameliorated by continued treatment with an SOD mimetic, tempol [31], suggesting that the PE-like symptoms of these model mice are precipitated by disruption of redox-regulated signaling during pregnancy. A further link between redox-regulated signaling and human pregnancy pathology has been provided by the most recently developed animal model for PE [32]. The homozygous deletion of Catechol-O-methyl transferase (*Comt*^-/-^) in pregnant mice results in the loss of 2ME2 which has direct involvement in redox-regulated signaling.

Despite the advantage of targeted disruption of a specific gene (*Comt*) in the mouse system and similarities in placental cell types in mouse and human, this animal model might be insufficient in understanding all aspects of the development of human preeclampsia and fetal growth restriction. The limitations of the mouse model for preeclampsia stem from intrinsic differences in the physiology of mouse and human pregnancies [33]. The mode of implantation [34,35] differs between mouse and human; there is shallow decidual invasion in mouse compared to extensive uterine remodeling by invasive trophoblasts in human. In addition, the uterine spiral arterioles in mice are remodeled by maternal factors rather than by invasive trophoblasts in human pregnancies [36], and the endocrine control of mouse pregnancy is mediated by a limited number of placental hormones compared to that of human [37]. Moreover, the short gestation period (3 weeks) in mice, the incomplete development and birth of altricial young are unlike human pregnancies [38]. All of these factors limit mouse pregnancy as a model system for human fetal growth restrictions.

### Hypothesis

2-methoxyestradiol 2 (2ME2) is a natural estradiol metabolite which induces microtubule depolymerisation, inhibits angiogenesis and is a promising anticancer drug by virtue of its ability to target leukaemia cells while sparing normal lymphocytes [39]. At first glance, the fact that the absence of the 2ME2 metabolite in mutant (*Comt*^-/-^) pregnant mice results in placental hypoplasia and vascular pathology to the extent that it leads to a preeclampsia-like phenotype [32], might appear surprising. However, a paradigm shift with recent discoveries [6] of redox-regulated signaling pathways for angiogenesis [10,17,18,40] are consistent with vascular pathology of 2ME2-deficient (*Comt*^-/-^) pregnant mice [32]. It should be emphasized that the temporal requirements for 2ME2 vary in normal pregnancies, its production being extremely low in early pregnancy during the peak period of angiogenesis and high later (third trimester) when placental development is complete [41,42].

Genetic and biochemical studies have established that 2ME2 is a potent inhibitor of MnSOD [43-45] and facilitates superoxide (O_2_^.-^) production [44]. In wild-type pregnant mice (*Comt*^+/+^), the inhibition of MnSOD at late pregnancy (dpc 12–14 in mouse, equivalent to peak activity of 2ME2 at 30–35 wks of gestation in human) would disengage NADPH-mitochondrial cross-talk by reducing critical H_2_O_2 _concentration. Lack of sufficient H_2_O_2 _will activate PHD (PHD-Fe^+2^) for hydroxylation of HIF-1α (HIF-1α.OH) and subsequent degradation [see Fig 1b and ref [25-29]]. This notion is in agreement with the established anti-angiogenic role of 2ME2 [46] and inhibition of HIF-1α in WT (*Comt*^+/+^) animals [32]. Such down-regulation of vascular remodeling and placental growth under normoxic conditions at late pregnancy by 2ME2 (peak activity at 30–35 wks of gestation), is presumed to be a physiological necessity to circumvent uncontrolled placental invasion which otherwise could potentially lead to pregnancy pathology. Premature induction of 2ME2 during early hypoxic growth (4–12 wks of gestation) would be detrimental to pregnancy, since the stability of HIF-1α in early hypoxic development is essential for vascular remodeling. Additionally, 2ME1 (2-methoxyestrone), an analogue of 2ME2 having no inhibitory effect on MnSOD [45], would fail to correct 2ME2 deficiency in *Comt*^-/- ^mice.

In *Comt*^-/- ^mice, the lack of inhibition of MnSOD under normoxic conditions (late pregnancy) would facilitate untimely accumulation of H_2_O_2 _which is essential for HIF-1α stability [32]. H_2_O_2 _would block hydroxylation of HIF-1α by inactivating PHD (PHD-Fe^+3^) [25-29] This view (Fig 1b) is supported by the observation that HIF-1α is labile following injection of 2ME2 in *Comt*^-/- ^mice at late pregnancy [32]. Increased accumulation of H_2 _O_2 _(> 200 μM/L) under normoxic conditions together with stable HIF1-α is sufficient to inflict vascular pathology in *Comt*^-/- ^mice. Moreover, HIF-1α is a potent mediator of myeloid cell (monocytes and macrophages) infiltration at the sites of inflammation [47] and lipopolysaccharide-induced sepsis [48]. Therefore stable HIF-1α alone at late pregnancy could elicit preeclampsia-like phenotypes in *Comt*^-/- ^mice.

### Testing the hypothesis

#### Oxidative stress as an inducer of preeclampsia in genetically normal mice

1. Preeclampsia symptoms in normal pregnant mice could be created by treating the animals with 2ME2. 2ME2 which peaks at third trimester of pregnancy, is a pro-oxidant and rather than an antioxidant as proposed by Kanasaki *et al *[32]. Therefore, 2ME2 could be detrimental to early hypoxic development. This hypothesis could be tested by daily injection of 2ME2 beginning at 3 days prior to pregnancy up to dpc 17 of gestation in genetically normal mice.

2. Since 2ME2 at low concentrations (0.3 mM) in combination with rotenone [44] is a potent inducer of O_2_^.-^, the same experiment could be repeated to ensure maximum oxidative effect. The logic behind using the combination is that rotenone at low concentration (50 nM) would direct O_2_^.- ^production by diverting electron flow from complex 1 of the electron transport chain to O_2_, while non-toxic concentrations of 2ME2 (0.3 μM) would synergistically facilitate O_2_^.- ^accumulation by inhibiting SOD [44].

#### Elimination of oxidative stress in mutant mice by antioxidants/activator of PHD

1. The proposed oxidative damage induced by accumulation of H_2_O_2 _in *Comt*^-/- ^mice could be rescued by treating the animals with synthetic MnSOD/catalase mimetics. Synthetic MnSOD/catalase mimetics have been shown to exhibit both SOD and catalase activities, and some are more potent, stable and cytoprotective than the native antioxidant enzyme SOD [49].

2. The preeclampsia phenotype in mutant animals could be rescued by treating the animals with ascorbate or specific activators of PHD (benzopyran or an inhibitor of diacylglycerol kinase, R59949).

### Implications of the hypothesis

While oxidative stress has been proposed to be central to placental pathogenesis and systemic vasculo-endothelial damage in human preeclampsia and a hypertensive mouse model [31], the concept has never been tested in genetically-defined animal models. The hypothesis and tests described here might contribute to the understanding of pathophysiologic sequences leading to the clinical manifestation of preeclampsia. Moreover, ROS are indispensible to angiogenesis, trophoblast differentiation, invasion and embryogenesis. The proposed experiments would help evaluate the importance of redox-regulated signaling in early as well as late pregnancy.

## Abbreviations

(ATP): Adenosine triphosphate; (GPCR): G protein-coupled receptor; (ISP3): Inositol triphosphate; (ER): Endoplasmic reticulum; (*Comt*): Catechol-O-methyl transferase; (2ME2): 2-methoxyestradiol 2; (HIF-1α): Hypoxia induciblefactor-1α; (SOD-1, 2 and 3): Superoxide dismutase 1–3; (MnSOD): Mitochondrial superoxide dismutase; (PHD): Prolylhydroxlases; (ROS): Reactive oxygen species; (WT): wild type.

## Competing interests

The authors declare that they have no competing interests.

## Authors' contributions

The authors discussed the clinical importance of different mouse models for preeclampsia and developed the hypothesis; SB wrote the manuscript. All authors read and approved the final manuscript.
